# Craniopharyngioma in a patient with acromegaly due to a pituitary macroadenoma

**DOI:** 10.4103/0256-4947.70581

**Published:** 2010

**Authors:** Hazem El-Bilbeisi, Mohammad Ghannam, Caramella F. Nimri, Azmi T. Ahmad

**Affiliations:** a From the National Center for Diabetes, Endocrinology, Genetics, Amman, Jordan; b From the Jordan University Hospital, Amman, Jordan

## Abstract

We present the first reported case of a craniopharyngioma as a second primary tumor in a patient with acromegaly due to a growth hormone (GH)-secreting pituitary adenoma. The patient was lost for follow-up for 18 years after trans-sphenoidal pituitary surgery for a GH-secreting pituitary adenoma. She presented with headaches and decreased visual acuity, and showed unsuppressed GH in an oral glucose load test with high IGF-1 levels. Brain MRI showed a suprasellar cystic mass and the patient underwent surgery for cyst drainage resulting in postoperative improvement in her vision. Biopsy of the mass confirmed the diagnosis of a craniopharyngioma. We stress the need for close follow-up of patients with acromegaly with adequate control of GH and IGF-1 levels.

Craniopharyngiomas are rare, mainly sellar/parasellar, epithelial tumors, arising along the path of the craniopharyngeal duct, with an as yet undescribed pathogenesis. Their overall incidence is around 0.13 cases per 100 000 person-years^1^ and they account for 2% to 5% of all primary intracranial neoplasms.^2^ Growth hormone (GH)-secreting pituitary adenomas are relatively rare, with a prevalence of 50 to 70 cases/million and an incidence of 3 to 4 new cases/million/year.^3^–^5^

The simultaneous occurrence of both a craniopharyngioma and a GH-secreting pituitary adenoma has not been reported previously in the literature. Cases of the coexistence of craniopharyngioma with prolactinoma^6^ or pineocytoma^7^ have been previously reported, but this combination is probably merely coincidental. With the rare exception of GH-releasing hormone (RH)-secreting gangliocytomas of the hypothalamus, GHRH-secreting carcinoids,^8^–^12^ and the even rarer GH-secreting islet cell tumors, GH hypersecretion is caused by an intrinsic pituitary neoplasm.

Two theories on the pathogenesis of craniopharyngiomas are that they arise from neoplastic transformation of embryonic squamous cell rests of the involuted craniopharyngeal duct,^13^ or that they result from metaplasia of adenohypophyseal cells in the pituitary stalk or gland.^14^,^15^ An interesting and noteworthy fact is that high levels of IGF-1 receptor expression have been demonstrated in cell lines and paraffin-embedded material in a subset of craniopharyngiomas. In fact, in this group of tumors, treatment with an IGF-1 receptor inhibitor caused growth arrest.^16^ However, further studies on a larger collection of cases are required to elucidate the clinical value of these results.

Acromegaly is known to be associated with high levels of IGF-1, which through activation of the IGF-1 receptor, results in cell proliferation and growth advantage, whereas the associated IGFBP3 bioactivity promotes an apoptotic advantage.^17^–^20^ Thus, excess GH, by inducing both IGFBP3 and IGF-1 levels, promotes dysregulated cell growth balance characterized by dynamic signals for cell apoptosis versus cell growth advantage. Having a subset of craniopharyngiomas expressing the IGF-1 receptor,^16^ but not the IGF-BP3 receptor, suggests that these cells would have a cell growth advantage induced by IGF-1 without the opposing apoptotic effect of IGFBP3, which might have been the case in our patient.

## CASE

**Figure 1:**
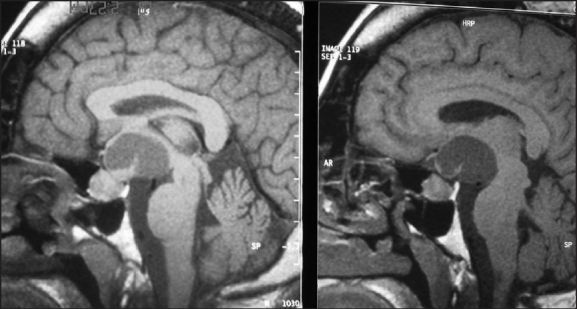
Sagittal MRI brain with enlarged pituitary and suprasellar cyst.

**Figure 2:**
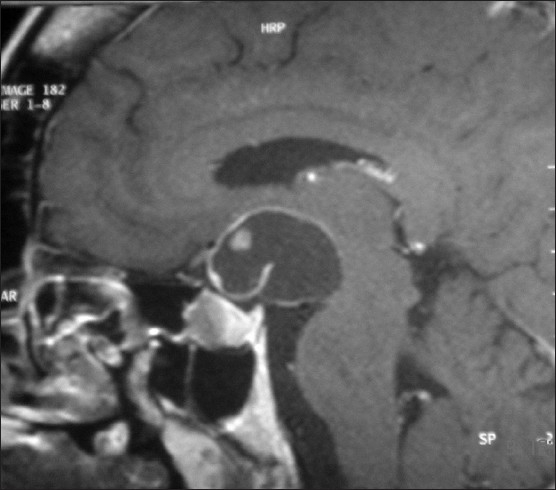
Sagittal MRI brain with enlarged pituitary and suprasellar cyst.

A 41-year-old woman presented to our university hospital emergency department in 2007 with complaints of occipital headache of increasing intensity and worsening blurred vision in her left eye for the last seven months prior to her visit. Recently, she had also noticed that her menstrual periods were becoming irregular. The patient had a history of acromegaly treated with trans-sphenoidal resection of a pituitary tumor in 1989. She was also known to have primary infertility for which she had not sought any medical help. She had been lost to follow-up since her surgery until this visit. On examination, she was found to have acromegalic features and a left eye temporal visual field defect on confrontation and visual field mapping. An x-ray of her skull revealed thickened calvaria with a wide, double-floored sella. An MRI of her brain revealed a multiloculated cystic mass seen in the suprasellar region with extension more to the left side, causing a mass effect on the surrounding structures without midline shift or hydrocephalus (**Figure 1,2**).

**Figure 3:**
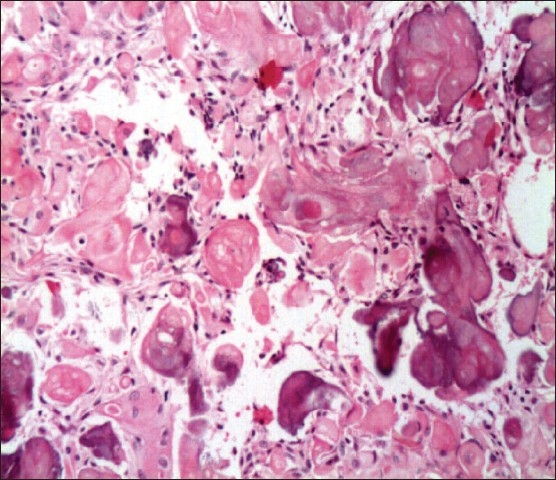
Craniopharyngioma anastomosing epithelial islands with palisaded layer of cells and a center of stellate cells along with nests of keratin (hematoxylin and eosin, ×20)

In the two months since her first visit, the patient had noticed a significant decrease in her left eye vision with an ability only to perceive light. On examination, her right eye was found to have a new temporal visual field defect. At that time, endocrine testing revealed hypogonadotropic hypogonadism with a serum prolactin level that was mildly elevated at 45 ng/mL. She was also found to have central hypothyroidism. Her GH was not suppressed even after an oral glucose load test. The baseline GH was 17.3 ng/mL; it became 13.0 ng/mL and 10.3 ng/mL at one and two hours. The IGF-1 level of 1006 ng/mL along with the unsuppressed GH confirmed the previous diagnosis of acromegaly. She underwent a left pterional craniotomy with subfrontal approach under microscopic guidance to obtain a tissue diagnosis and drain the cystic tumor. She developed transient diabetes insipidus postoperatively, but her vision improved significantly following surgery. She received perioperative stress doses of steroids, which were tapered rapidly afterwards. The patient was also started on L-thyroxine replacement therapy. Tissues obtained from surgery confirmed the diagnosis of a craniopharyngioma (**Figure 3**). Six months after presentation, she was still being followed up at the endocrine and the neurosurgery clinics for further management.

## DISCUSSION

To our knowledge, this is the first reported case of a craniopharyngioma developing in a patient with a GH-secreting pituitary adenoma. A craniopharyngioma comprises only 3% of intracranial neoplasms with a tendency to affect children and young adults. 21 Headache and visual field defects are the most common presenting clinical features (64% and 55%, respectively). Craniopharyngiomas can result in a high incidence of deficiencies of GH, luteinizing hormone/follicle-stimulating hormone, thyroid-stimulating hormone, and adrenocorticotropic hormone (>80%), and diabetes insipidus (65%).^22^

Both a craniopharyngioma and the pituitary gland share the same embryonic origin. They arise from the Rathke pouch, which is a diverticulum of the roof of the embryonic oral cavity.^23^ A developmental mishap or a genetic abnormality might explain the presence of these two different tumors in the same individual. A previous study attempted to implicate the beta-catenin gene in the initiation and subsequent growth of craniopharyngiomas and pituitary adenomas. Several mutations of the beta-catenin gene have been found in adamantinomatous craniopharyngiomas, but not in pituitary adenomas.^24^

The collision lesions of the sellar region (two simultaneous tumors or cysts in the perisellar region) have been previously described in case reports or small case series. The most common presentation of collision sellar lesions is the presence of a simultaneous pituitary adenoma with a Rathke cyst. A previous case report exists of simultaneous GH-secreting pituitary micro-adenoma with a Rathke cleft cyst, causing compression of the optic chiasm with resultant visual field defects.^25^ Adenomas in this situation usually secrete prolactin.^26^ This is probably due to stalk compression from the cyst with resultant lactotroph hyperplasia. There is one case report of a craniopharyngioma with a gonadotrophin-secreting pituitary adenoma, but this occurrence remains very rare.^27^

In a study of the nationwide Swedish cancer family registry, meningioma was reported as the most frequent second primary tumor in individuals with a previous diagnosis of a first primary tumor of pituitary or parathyroid adenomas.^28^ In a group of patients with pituitary adenomas who underwent surgery and radiotherapy, meningioma was the most common second primary intracranial neoplasm at 10 to 20 years of follow-up.^29^ Craniopharyngioma was not reported in this series of more than 400 patients. Few case reports exist of simultaneous pituitary adenomas and meningiomas, either in the parasellar region or the fourth ventricle without any previous history of radiotherapy.^30^,^31^

Acromegaly has been arguably associated with an increased risk for several tumors with the strongest association reported with colorectal carcinoma.^32^ Increasing evidence from in vitro data, animal studies, and studies in nonacromegalic patients continue to strengthen the role of the GH/IGF-1 axis in tumor development. In addition to the classical endocrine actions of the GH/IGF-I axis, important paracrine and autocrine effects of locally produced GH and/or IGF-I are being increasingly recognized.^33^

Our patient had evidently suffered from uncontrolled acromegaly for several years with elevated GH and IGF-1 levels. Given the close anatomic proximity of the GH-secreting pituitary macroadenoma and the suprasellar craniopharyngioma in this patient, it would be reasonable to assume that the paracrine effects of the GH/IGF-1 axis would have been exaggerated. There is a paucity of literature investigating the tumorigenic effects of the GH/IGF-1 axis on brain tumors and craniophayngiomas, in particular, due to the rarity of this occurrence. Though rare, this complication of acromegaly might have been potentially prevented or detected earlier if the patient had maintained closer follow-up after her trans-sphenoidal pituitary resection. Better biochemical control of her acromegaly might have prevented the emergence of the craniopharyngioma. It is debatable whether the patient’s clinical picture and biochemical evidence of acromegaly were the result of GHRH secretion from the suprasellar craniopharyngioma. A possible scenario is that she had a craniopharyngioma at time of initial presentation, which was too small to be detected by the radiological studies done at that point of time. However, we have no proof that this was the case. Additionally, if the GH excess was the result of GHRH, it would have resulted in pituitary GH-secreting cell hyperplasia, rather than a distinct pituitary adenoma, which is the case in our patient. She first underwent trans-sphenoidal surgery for the GH-secreting pituitary adenoma, which did not result in adequate control of her disease. After an interval of several years without follow-up, she presented with the second pathology. It is worth noting that in a series of 121 cases of craniopharyngioma in children, none presented with acromegaly. In fact, craniopharyngioma mostly presents with hormone deficiency syndromes, with the sole exception of the expected elevation in serum prolactin levels.^22^ In fact, there were no previous reported cases of any GHRH-secreting craniopharyngioma in a Medline search. Unfortunately, we were not able to obtain serum GHRH levels in this patient due to the unavailability of this assay in any of the local hospitals and laboratories.

In summary, we report a patient with uncontrolled acromegaly who presented with headache and visual field defects due to a suprasellar craniopharyngioma. She had central hypothyroidism and hypogonadotrophic hypogonadism. To our knowledge, this is the first reported case in the literature.
